# High Nutritional Risk Is Associated with Poor Functional Status and Prognostic Biomarkers in Stroke Patients at Admission to a Rehabilitation Unit

**DOI:** 10.3390/nu15194144

**Published:** 2023-09-25

**Authors:** Olivia Di Vincenzo, Ermenegilda Pagano, Mariarosaria Cervone, Raffaele Natale, Annadora Morena, Alessandra Esposito, Fabrizio Pasanisi, Luca Scalfi

**Affiliations:** 1 Department of Public Health, School of Medicine, Federico II University, via Sergio Pansini 5, 80131 Naples, Italy; scalfi@unina.it; 2 Casa di Cura Santa Maria del Pozzo Hospital, via Pomigliano 40, Somma Vesuviana, 80049 Naples, Italy; gildapagano3@gmail.com (E.P.); marycervone@virgilio.it (M.C.); nutrizionistaalessandra@gmail.com (A.E.); 3 Department of Clinical Medicine and Surgery, School of Medicine, Federico II University, via Sergio Pansini 5, 80131 Naples, Italy; raffaelenatale1993@gmail.com (R.N.); morena.annadora@gmail.com (A.M.); pasanisi@unina.it (F.P.)

**Keywords:** malnutrition, GNRI, PNI, CONUT, inflammation, older patients

## Abstract

Considering that malnutrition (undernutrition) is common in stroke patients and may negatively impact body function, the aim of this study was to determine the relationship between nutritional risk and functional status in stroke patients at admission to a rehabilitation unit. Nutritional risk was assessed using the Geriatric Nutritional Risk Index (GNRI), the Prognostic Nutritional Index (PNI) and the Controlling Nutritional Status (CONUT) score. Functional status was assessed using the Barthel Index, the modified Rankin Scale, the Trunk Control Test and the Sitting Balance Scale, and cognitive function was assessed using the Short Portable Mental Status Questionnaire. C-reactive protein, fibrinogen and D-dimer were also evaluated as established prognostic biomarkers. Stroke patients (n = 245; age 69.7 ± 12.8 years; 47%, women; 82% ischemic stroke) at admission to a rehabilitation unit were included in this study. A high prevalence of nutritional risk was detected with each tool and was found to be greater using the GNRI and in patients aged ≥75 years. Multiple logistic regression analysis showed that age and dysphagia were independent predictors of high nutritional risk. High risk groups performed worse on all functional tests compared to the low-risk groups (*p* < 0.05). Nutritional risk with each tool was associated with functional and cognitive statuses (with the highest correlation being with the Trunk Control Test). Significant associations were also found with C-reactive protein, fibrinogen and D-dimer. In conclusion, a high nutritional risk, as evaluated with the GNRI, the PNI and the CONUT score, was detected in stroke patients at admission to a rehabilitation unit. High nutritional risk was associated with functional status and with predictors of clinical outcomes (and specifically in older patients).

## 1. Introduction

Stroke is a cardiovascular disease which represents not only a leading cause of death worldwide [1] but also a major cause of temporary or chronic disability, with important consequences in social terms and costs [2,3]. The persistence of a condition of severely impaired motor functioning after rehabilitation seems to anticipate the risk of mortality in the following years [4].

As defined by the European Society for Clinical Nutrition and Metabolism (ESPEN) [5], malnutrition (undernutrition) can be defined as “a state resulting from lack of intake or uptake of nutrition that leads to altered body composition (decreased fat-free mass) and body cell mass with diminished physical and mental function and impaired clinical outcome from disease”. As a consequence, undernutrition negatively affects diagnosis, prognosis and the clinical course of various acute or chronic diseases [6].

Undernutrition is a common feature in stroke survivors (in most cases, older individuals) and is due to the interactions of different factors, such as comorbidities, dysphagia, etc.; it is frequently undiagnosed and untreated and has a prevalence ranging from 3 to 87%, which increases from the acute to the post-acute phase [3]. Malnutrition has been shown to worsen quality of life, as well as to be negatively associated with several clinical outcomes in both the short- and the long-term periods, such as mortality, functional recovery, infections and length of stay [7]. 

Nutritional screening is the process used to identify patients at nutritional risk (i.e., risk of malnutrition) or who may be at risk. An early nutritional screening represents the first step of the nutritional care of patients [5,8,9,10] but is often not adequately considered in the multidisciplinary approach to the diagnosis and management of patients affected by acute or chronic diseases. A recent literature review [6] indicates that various nutritional screening tools have been used in stroke patients (upon admission to hospital, during hospital stay or during rehabilitation) with a varying prevalence of nutritional risk, possibly because of the tool used and the clinical setting. Specifically, Geriatric Nutritional Risk Index (GNRI), Prognostic Nutritional Index (PNI) and Controlling Nutritional Status (CONUT) score have become popular because of the ease of retrieving laboratory test data (i.e., serum albumin, lymphocyte count) [11]. GNRI and PNI [6], as well as CONUT score, have been demonstrated to be independently associated with a higher risk of mortality and poor functional outcome both in the hospital and after discharge [7].

The evaluation of the functional status and selected laboratory tests also represents a major part of the comprehensive assessment of stroke patients; functional status (for instance, independence in activities of daily living, mobility and balance) is commonly evaluated using different scales while parameters such as C-reactive protein (CRP), fibrinogen and D-dimer have been identified as prognostic biomarkers [12,13].

Assessing whether a high nutritional risk is related to poor functional status might improve the clinical assessment in the short and long term. So far, evidence has been reported for the association between nutritional risk and some scales of functional status in acute stroke patients or in the long term, whereas very sparse data are available regarding this association at admission to rehabilitation facilities [6,7].

Against this background, the present study aimed to assess nutritional risk using the GNRI, the PNI and the CONUT score and to relate nutritional risk with functional status, cognitive status and selected prognostic biomarkers in a cohort of stroke patients referred to a rehabilitation unit after hospital discharge.

## 2. Materials and Methods

### 2.1. Study Design and Patients

This single-center retrospective cross-sectional study involved consecutive patients with stroke admitted to a neuromotor rehabilitation unit (S. Maria del Pozzo Hospital, Somma Vesuviana, Naples, Italy) following hospital discharge, from January 2021 to June 2023.

The inclusion criteria were as follows: (a) patients with a diagnosis of ischemic or hemorrhagic stroke (including subarachnoid hemorrhage), documented using magnetic resonance imaging or computed tomography; (b) age ≥60 years; (c) time since the stroke onset less than one month. Patients with cognitive disorders interfering with understanding and signing informed consent were excluded.

The study was conducted following the ethical principles of the 1964 Declaration of Helsinki and was approved by the Campania Sud Ethics Committee (Italy) (approval number 147/2023). Informed consent was obtained within 48 h from admission. The study staff members were all trained, according to the good clinical practice guidelines, with theoretical and practical sessions, to perform the screening tests, the functional tests and other measurements.

### 2.2. Clinical and Functional Assessment

Demographic and anamnestic clinical data were recorded at admission while functional assessment was carried out within 48 h from admission.

Based on the collected medical information, a diagnosis of ischemic stroke, hemorrhagic stroke or subarachnoid hemorrhage was made. Comorbidities, including atrial fibrillation, hypertension, diabetes mellitus, coronary heart disease and hyperlipemia were evaluated at admission according to routine diagnosis process. Previous stroke was recorded according to the description by the patient or family members. Dysphagia or swallowing ability was assessed using the Food Intake LEVEL Scale (FILS) [14] which is a 10-point scale for the level of feeding and swallowing status with a score of 1–3 (no oral intake), 4–6 (combined oral intake and alternative nutrition), 7–9 (oral intake only) and 10 (normal).

Functional status of patients was evaluated using different scales.

The Barthel Index (BI) assesses independence in activity of daily living related to mobility and self-care activities. It includes 10 items, and the total score is between 0 (fully dependent) and 100 (fully independent) [15].

The modified Rankin Scale (mRS) measures the level of disability. Each patient is categorized into one of the six possible categories ranging from 0 (no symptoms) to 6 (death) [16].

The Trunk Control Test (TCT) assesses rolling, sitting and maintaining balance in the sitting position. Each item allocated 0 to 25 points; the possible total score is 100 with a higher score indicating a better performance [17].

The Sitting Balance Scale (SBS) evaluates static and dynamic sitting balance, ranging from 4 = normal (able to perform the test without any physical assistance) to 1 = poor (unable to maintain a static position) [18].

Finally, cognitive status was assessed using the Short Portable Mental Status Questionnaire (SPMSQ), which is calculated by adding the number of incorrect responses (0 = correct), (1 = incorrect) for each of the fields (orientation, knowledge and working memory). The total score is 10 and patients unable to answer because of aphasia were scored as giving an incorrect answer. Higher scores indicate more severe cognitive impairment [19].

### 2.3. Biochemical Variables

Routine venous blood samples were taken within 24 h from admission. The following variables related to nutritional status and inflammation were selected: albumin, cholesterol, lymphocyte count, neutrophil count, haemoglobin, platelet count, C-reactive protein, fibrinogen and D-dimer.

### 2.4. Anthropometry

The measurements were performed during the first 48 h after admission by the same staff members to reduce the risk of bias following standard procedures according to Lohman et al. [20]. Body weight was measured in duplicate to the nearest 0.1 kg using an extra-large platform scale, with the patient in a wheelchair wearing light clothes and no shoes (7708 platform scale; Soehnle Industrial Solutions GmbH, Backnang, Germany). Stature was measured in triplicate to the nearest 0.1 cm in the supine position with the patient lying in bed using a portable stadiometer (Seca 213; Seca Hamburg, Germany). Mean values were used for calculating body mass index (BMI) as body weight (kg) divided by squared stature (m^2^).

### 2.5. Nutritional Risk Assessment

Nutritional risk was assessed with three different screening tools.

GNRI was calculated with the following formula: GNRI = 14.89 × serum albumin (g/dL) + 41.7 × (body weight [kg]/ideal body weight [kg]) [6]. Ideal body weight was defined according to the Lorentz formula: for men, (stature—100)—((stature—150)/4); for women, (stature—100)—((stature—150)/2). The actual body weight/ideal body weight ratio was regarded as 1 when the actual body weight exceeded the ideal body weight [21]. According to the original paper, patients were classified as at normal (score ≥98), mild (score 97–92), moderate (score 91–82) or severe (score <82) nutritional risk.

PNI was calculated according to the following formula: PNI = 10 × serum albumin (g/dL) + 0.005 × total lymphocyte count (n/mm^3^) [6]. According to the original paper, a score >38 reflects a normal nutritional status, with scores of 35–38 and <35 indicating moderate and severe nutritional risk, respectively. In this case, there is no “mild/light” category.

CONUT score was determined from serum albumin concentration, total peripheral lymphocyte count and total cholesterol concentration according to the extent of decrease compared to the normal ranges (possible score 0–2–4–6 for albumin, 0–1–2–3 for both lymphocyte count and total cholesterol). Based on total score, patients were classified as at normal (score 0–1), light (2–4), moderate (5–8) or severe (9–12) nutritional risk [6].

Overall, a high nutritional risk was defined as GNRI < 92, PNI ≤ 38 or CONUT score ≥ 5.

### 2.6. Statistical Analysis

To our knowledge no previous studies have investigated the association between nutritional risk and functional status and selected biochemical parameters in stroke patients at admission to rehabilitation. Therefore, in a post hoc analysis, statistical power was 0.80 for r = 0.20 (correlation) and alpha level = 0.05, with a sample size = 194 participants. Data analysis was conducted using the SPSS Statistics software (version 27.0.0, SPSS Inc., Chicago, IL, USA). Nutritional risk was evaluated using GNRI, PNI and CONUT score. All tests were 2-tailed, and a *p* value <0.05 was considered statistically significant. The Kolmogorov–Smirnov Test and the Shapiro–Wilk Test were used as tests of normality to examine whether variables were normally distributed. For descriptive analysis, patients’ characteristics were reported as mean plus standard deviation, median plus interquartile ranges or frequencies (percentages). Comparisons between groups were performed with the χ^2^ test or Fisher exact test, the Student *t* test or the Mann–Whitney U test, as appropriate. Partial correlation analysis was performed using Spearman’s rank coefficient, to assess the relationship between GNRI, PNI and CONUT score and age, BMI, BI, mRS, SBS, TCT, SPMSQ, CRP, fibrinogen and D-dimer. Univariate and multivariate analyses with logistic regression were used to determine independent risk factors of nutritional risk (for each tool) in stroke patients, considering subjects with GNRI <92, PNI ≤38 and CONUT score ≥5 as high-risk groups. Potential predictors were age, sex, BMI, dysphagia, atrial fibrillation, hypertension, diabetes mellitus, heart disease, hyperlipemia, previous stroke, type of stroke and time from stroke onset.

## 3. Results

During the study period, 259 stroke patients were admitted to the rehabilitation unit. Of these patients, 10 were excluded from the analysis due to insufficient data, 2 patients failed to provide informed consent and 2 refused to participate. Therefore, a total of 245 post-acute stroke patients (47% women; 81.6% ischemic, 17.2% hemorrhagic and 1.2% with subarachnoid hemorrhage) met the inclusion criteria and were ultimately included in this study, with a mean age of 69.7 ± 12.8 years (range 60–96). Patients’ general and clinical characteristics are detailed in Table 1. Men had a greater weight, stature and BMI than women (*p* < 0.05) and similar age. Overall, 1% of patients were underweight, 26% normal weight, 51% overweight and 22% obese. Only five patients were treated with statins (no one with low cholesterol level). A high prevalence of hypertension (78%) was found in both sexes whereas a greater prevalence of atrial fibrillation was observed in women compared to men (Table 1). 

Data on functional assessment showed that women reported worse scores than men on most scales (*p* < 0.05), except for mRS (Table 1). Taking into account the laboratory parameters, 69% of patients showed low serum albumin levels (<3.5 g/dL); 77% low cholesterol (<180 mg/dL); 58% low lymphocyte count (<1600 cells/mL); 49% low hemoglobin (<13 g/dL); 72% high CRP (>5 mg/L); 65% high fibrinogen (>450 mg/dL); and 69% high D-dimer (>0.5 μg/mL).

**Figure 1:**
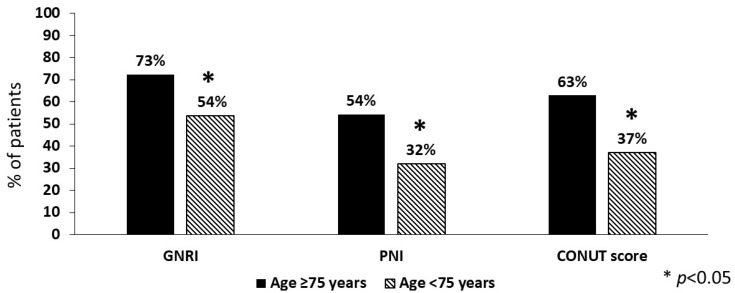
Prevalence of high nutritional risk in stroke patients aged ≥75 years compared to the younger ones (n = 105 and n = 140, respectively). GNRI = geriatric nutritional risk index; PNI = prognostic nutritional index; CONUT = controlling nutritional status score.

Concerning the nutritional risk, women had a lower median value of GNRI than men (*p* < 0.001) but similar PNI and CONUT score. Overall, the prevalence of high nutritional risk was greater for GNRI (62%) than for PNI (42%) or CONUT score (48%). In addition, a mild/light risk of malnutrition was detected in 38% and 23% of patients according to GNRI and CONUT score, respectively. Notably, 33% of patients had no risk at all (0 tools), while in 37% of them a high nutritional risk was detected by all the tools (10% with two tools; 20% with one tool). As shown in Figure 1, nutritional risk was much more prevalent in patients aged ≥75 years (n = 105) vs. the younger ones (n = 140) with each of the tools, again with higher prevalence for GNRI.

Multiple logistic regression analysis showed that in addition to age and sex (only for GNRI), dysphagia emerged as an independent predictor of high nutritional risk according to GNRI (OR 2.50, 95% CI 1.24–4.65, *p* = 0.004), PNI (OR 1.98, 95% CI 1.12–3.50, *p* = 0.019) or CONUT score (OR 2.15, 95% CI 1.20–3.83, *p* = 0.010), while there was no significant effect due to type of stroke, time from stroke onset, BMI, atrial fibrillation, hypertension, diabetes mellitus, heart disease, hyperlipemia and previous stroke.

Table 2 shows variables of interest according to high and low nutritional risk. Patients with high nutritional risk were older compared to the ones at low risk according to the three tools.

A very high prevalence of hypertension was found independently of nutritional risk whereas a significantly higher prevalence of atrial fibrillation was found in the high-risk compared to the low-risk groups (GNRI and PNI) (Table 2). Notably, the prevalence of dysphagia was higher in the high-risk than the low-risk groups (*p* < 0.001) and in patients aged ≥75 years compared to the younger ones.

Data on functional assessment (Table 2) showed that patients at high nutritional risk performed worse on all functional scales, and that was true also for cognitive assessment (*p* < 0.05).

Table 3 shows the association between nutritional risk and functional status. Overall, associations were significant for all the tests selected with the highest correlation being between TCT and each tool. When considering stroke patients aged ≥75 years (n = 105) (Table 4), significant but weak associations were observed for some functional tests.

As shown in Table 2, considering the laboratory parameters included in one or more of the three screening tools, serum albumin (by GNRI, PNI and CONUT score), total cholesterol (by CONUT score) and lymphocyte count (by PNI and CONUT score) were lower in high vs. low nutritional risk group. A significant difference also emerged for hemoglobin by all the tools.

**Figure 2:**
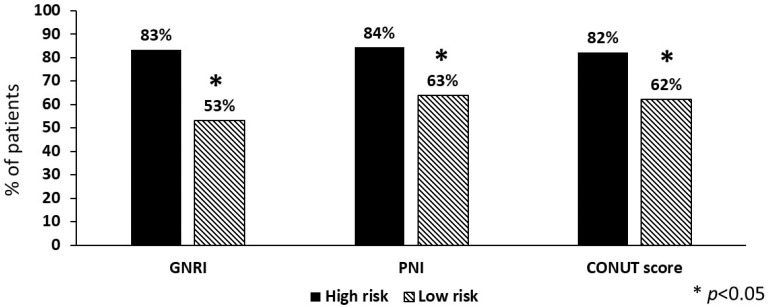
Prevalence of high levels (>5 mg/L) of C-reactive protein in stroke patients (n = 245) according to nutritional risk. GNRI = geriatric nutritional risk index; PNI = prognostic nutritional index; CONUT = controlling nutritional status score.

Taking into account laboratory parameters, the values of CRP, fibrinogen and D-dimer were significantly greater in the high-risk compared to the low-risk group for each of the three tools considered (Table 2). CRP >5 mg/L was more prevalent in the high-risk compared to the low-risk group (GNRI 83% vs. 53%, PNI 84% vs. 63%, CONUT score 82% vs. 62%), and the same was true for D-dimer >0.5 μg/mL (GNRI 89% vs. 50%, PNI 91% vs. 61%, CONUT score 86% vs. 62%), as shown in Figure 2.

On the other hand, fibrinogen >450 mg/dL was similar in the high-risk compared to the low-risk group (GNRI 70% vs. 63%, PNI 70% vs. 65%, CONUT score 70% vs. 65%). In addition, significant associations were observed between nutritional risk and CRP, fibrinogen and D-dimer (Table 3) which were even higher (CRP and fibrinogen) in patients aged ≥75 years (Table 4).

Finally, patients at low risk of malnutrition according to all three tools compared to the remaining ones were younger (*p* < 0.001) and had lower weight and stature, the best scores in all the functional scales (*p* < 0.001) and the lowest CRP, fibrinogen and D-dimer levels (*p* < 0.001).

## 4. Discussion

This study evaluated nutritional risk (using three different tools) and its relationships with functional status in stroke patients admitted to a rehabilitation unit. A high prevalence of nutritional risk was observed for each of the tools and was found to be greater using GNRI and in patients aged ≥75 years. Nutritional risk was significantly associated with functional and cognitive status and also with predictors of clinical outcomes such as CRP, fibrinogen and D-dimer.

Malnutrition is common in stroke patients and is negatively related to poor clinical outcomes, in both the short- and long-term period, wherein the foremost concerns are disability and mortality [7]. In addition, it should be mentioned that nutritional status deteriorates during hospitalization due to low food intake, inflammation, complications, comorbidities, etc. [2,3,6,22]. Thus, in the clinical setting, nutritional screening should play an important role in the multidisciplinary approach to the management of stroke patients. GNRI, PNI and CONUT score have become very popular and frequently used in the last few years in stroke patients [6], being, in different ways, based on simple information on weight, albumin, lymphocyte count and total cholesterol. Overall, nutritional risk has been assessed more frequently in the acute phase of stroke and less frequently in the rehabilitation setting; in these latter patients, according to a recent review [6], few studies indicated that the prevalence of nutritional risk was about 27% [23,24] and 75% [25] for GNRI and 14% for PNI [4], with no information available for CONUT score. In addition, GNRI (nine studies), PNI (one study) and CONUT score (one study) have being related to outcomes such as mortality, functional impairment, infection and dysphagia [6].

The present study provides novel data on the use of different nutritional screening tools in stroke patients admitted to a rehabilitation unit. Nutritional risk was high for each of the tools used, in line with previous studies [6], being more prevalent when using GNRI than CONUT and PNI, and it is significantly greater in the male sex for GNRI and in older patients (age ≥75 years). 

Diabetes mellitus, hypertension and history of previous stroke are associated with malnutrition in hospitalized stroke patients [2]; moreover, the presence of dysphagia has proven to be a major risk factor for developing malnutrition and complications during hospitalization [2]. In the group of patients we studied, nutritional risk (for each of the three screening tools) was negatively influenced by sex, age and dysphagia, whereas BMI, atrial fibrillation, hypertension, diabetes mellitus, heart disease, hyperlipemia, previous stroke, type of stroke and time from stroke onset were not found to be prognostic factors. This finding is in line with recent papers showing that younger age and hyperlipemia were independently associated with lower CONUT scores [26] and that dysphagia was associated with high nutritional risk according to GNRI [25,27,28,29]. Although diabetes is a nutritional disorder that might negatively affect the clinical outcomes in stroke patients, it does not seem to have an effect on the nutritional risk in the patients we studied. Indeed, it should be noted that dysphagia was more prevalent in patients with diabetes.

As a next step, variables of interest were compared between high and low nutritional risk groups. Physical impairment after stroke is mostly considered as the consequence of brain injury and lateralized paralytic injury [30]; indeed, malnutrition has been significantly associated in hospitalized patients with functional status at discharge and later [7,31], whereas, so far, poor evidence is available for the rehabilitation setting [6].

As new information in the present study, well-recognized tests for evaluating functional and cognitive status were significantly associated with nutritional risk in patients admitted to a rehabilitation unit. At first glance, the differences between median values were more marked using GNRI; this perception is reinforced by the stronger correlations observed between GNRI and functional tests compared to PNI and CONUT score. As another novel insight, there was a significant association between nutritional risk and cognitive impairment; previously, only one study [32] found that low GNRI was independently associated with cognitive impairment 3 months after stroke.

Selected laboratory tests were also compared between high and low nutritional risk groups. First, there are expected differences for those parameters that are included in the tools; in other words, albumin differed between high-risk vs. low-risk groups for each of the three tools; lymphocyte count for the PNI and CONUT score; and total cholesterol only for the CONUT score.

Furthermore, this study focuses specifically on laboratory parameters, such as CRP, fibrinogen and D-dimer, that have been identified as prognostic biomarkers in stroke patients [12,13], being related to inflammation, fibrinolysis, thrombosis and more in general to complex tissue response to ischemia/hemorrhage [12,13]. Of note, the combination of different biomarkers might greatly improve the accuracy of a stroke outcome prediction [13].

For the first time, the findings of the present study show that for CRP, fibrinogen and D-dimer, very significant differences were observed between low-risk and high-risk patients. It should be noted that CRP > 5 mg/L was more prevalent in the high-risk vs. the low-risk groups with each tool, with the highest difference for the GNRI. Also, significant associations were found between nutritional screening and CRP, fibrinogen and D-dimer.

A further point to be addressed is the effect of the type of stroke and older age on nutritional risk and its relationships with the aforementioned variables of interest.

Since just 17.2% of patients had suffered from hemorrhagic stroke, only some preliminary observations may be made, taking into account that results in ischemic patients, as expected, were comparable to the ones for the group as a whole. The prevalence of nutritional risk in hemorrhagic patients was similar to that of the group as a whole. In addition, in a multivariate analysis, the relationships between nutritional risk and functional status were not affected by the type of stroke. It is worth noting that nutritional risk has been rarely evaluated in hemorrhagic patients in previous studies [6] and that specifically designed studies are needed to assess the differences in nutritional status between ischemic and hemorrhagic stroke patients.

Finally, we specifically looked at patients aged ≥75 years, who are expected to exhibit a higher prevalence of nutritional risk and an impaired physical function due not only to physiological age-related changes [33,34] but also because of alterations to appetite/feeding and increased protein requirements after stroke [2]. The present study showed that the nutritional risk strongly increased with age, with the highest prevalence detected with the GNRI. Also, functional but not cognitive assessment was significantly associated with nutritional risk. In addition, the relationships between nutritional risk and the selected biomarkers were greater (especially for CRP), compared to the whole sample.

To our knowledge, this is the first study providing data on the use of different nutritional screening tools in the same cohort of stroke patients at admission to rehabilitation. Functional and cognitive status was assessed using standard scoring systems, and major biochemical prognostic factors were selected. 

Nevertheless, there are some limitations that need to be addressed. First, this was a single-center study which limited the generalization of the results. Second, due to the low number of participants with hemorrhagic stroke, a detailed analysis on hemorrhagic patients was not possible. No information is available regarding the diet habits or the nutritional status of the patients before stroke which, in theory, may affect neurological recovery [35]. Also, information on other parameters of nutritional status, for instance body composition, was not taken into consideration. Other biochemical parameters or tools to assess functional and cognitive status, not available for the present retrospective studies, may also be related to nutritional risk. Further prospective studies are needed to investigate variations in the nutritional risk and functional status following rehabilitation.

## 5. Conclusions

This study contributes novel findings to the comprehensive assessment of stroke patients admitted to rehabilitation. A high nutritional risk (assessed with the GNRI, the PNI and the CONUT score) was detected in patients at rehabilitation after acute stroke. Furthermore, age and dysphagia emerged as independent predictors of high nutritional risk according to each screening tool. Additionally, a high nutritional risk was associated with worse functional and cognitive status and higher inflammation after acute stroke. These findings were confirmed also by considering older patients.

This study suggests that nutritional risk should be assessed regularly in patients at admission to rehabilitation after stroke for a more comprehensive identification of patients at higher risk of poor functional capacity. Indeed, further studies are required to evaluate changes in the nutritional risk and functional status subsequent to rehabilitation.

## Figures and Tables

**Table 1:** Patients’ general, functional and clinical characteristics by sex.

	Total	Men	Women	*p*
	n = 245	n = 130	n = 115	
Age, years	69.7±12.8	68.4±12.3	71.0±13.2	0.053
Weight, kg	70.2±12.5	76.8±11.7	68.9±14.8	<0.001
Stature, cm	164.0±9.7	169.7±7.6	158.0±8.1	<0.001
BMI, kg/m²	26.9±4.2	26.3±3.0	27.5±5.1	<0.001
**Stroke risk factors**	n(%)	n(%)	n(%)	
Atrial fibrillation	41(16.7)	14(10.8)	27(23.5)	<0.01
Hypertension	192(78.4)	97(74.6)	95(82.6)	0.162
Diabetes mellitus	87(35.5)	46(35.4)	41(35.7)	1.000
Coronary heart disease	92(37.6)	47(36.2)	52(40.0)	0.429
Hyperlipemia	107(43.7)	53(40.8)	54(57.0)	0.367
Previous stroke	39(15.4)	21(16.2)	18(15.7)	1.000
**Dysphagia**	94(38.8)	44(33.8)	50(44.6)	0.112
**Functional assessment**				
BI	5[5–15]	5[5–15]	5[0–10]	<0.01
mRS	4[4–5]	4[4–5]	4[4–5]	0.122
TCT	24[0–48]	36[12–48]	12[0–36]	<0.01
SBS	2[1–3]	2[2–3]	2[1–3]	<0.01
SPMSQ	6[4–10]	5[2–8]	8[5–10]	<0.001
**Laboratory parameters**				
Albumin, g/dL	3.19±0.50	3.29±0.48	3.07±0.50	<0.01
Cholesterol, mg/dL	145.4±40.1	141.4±39.3	153.0±41.6	<0.01
Lymphocyte count/mL	1400[1000–1800]	1300[1000–1800]	1400[975–1900]	0.266
Neutrophil count/mL	5500[4300–7200]	5600[4300–6950]	5400[3950–7550]	0.631
Hemoglobin, g/dL	12.8±1.54	13.2±1.68	12.4±1.37	<0.001
Platelet count/mL × 1000	245[197–309]	235[180–307]	252[212–309]	0.229
C-reactive protein, mg/L	16.2[4.6–46.2]	15.1[4.4–47.7]	16.3[4.8–52.8]	0.481
Fibrinogen, mg/dL	544±164	531±148	540±185	0.639
D-dimer, μg/mL	1.13[0.55–2.44]	1.13[0.49–2.20]	1.13[0.59–3.04]	0.771
**Nutritional risk screening tools**				
GNRI	89[84–94]	90[85–95]	88[83–92]	<0.001
PNI	39[34–43]	40[35–44]	37[34–43]	0.179
CONUT score	5[3–7]	4[3–7]	5[3–7]	0.286

Continuous variables were expressed as mean ± SD or median [interquartile range]. Categorical variables are expressed as frequencies (percentages). BMI = body mass index; BI = Barthel index; mRS = modified rankin scale; TCT = Trunk control test; SBS = sitting balance scale; SPMSQ = short portable mental status questionnaire; GNRI = geriatric nutritional risk index; PNI = prognostic nutritional index; CONUT = controlling nutritional status score.

**Table 2:** General, functional and clinical characteristics of the 245 patients by risk of malnutrition.

	GNRI			PNI			CONUT Score		
	High Risk, n *=* 151	Low Risk, n = 94	*p*	High Risk, n *=* 102	Low Risk, n = 143	*p*	High Risk, n *=* 118	Low Risk*,* n *=* 127	*p*
n (%) women	81(53.6)	34(36.2)	*	56(54.9)	59(41.3)	*	58(49.2)	57(44.9)	
Age, years	72.6±11.9	66.3±12.3	#	73.8±11.3	67.0±12.5	#	73.9±11.0	66.2±12.7	#
Weight, kg	71.0±13.4	75.4±13.0	§	70.0±14.3	74.7±12.2	§	70.5±13.4	74.7±13.3	*
Stature, cm	162.5±9.9	165.5±9.5	§	161.5±10.2	165.2±9.2	§	162.210.1	165.0±9.4	§
BMI, kg/m²	26.6±3.9	27.0±3.5		26.6±4.1	26.7±3.6		26.6±3.8	27.0±3.8	
**Stroke risk factors**	n(%)	n(%)		n(%)	n(%)		n(%)	n(%)	
Atrial fibrillation	31(20.5)	10(10.6)	*	23(22.5)	18(12.6)	*	23(19.5)	18(14.2)	
Hypertension	121(80.1)	71(75.5)		83(81.4)	109(76.2)		95(80.5)	97(76.4)	
Diabetes mellitus	56(37.1)	31(33)		41(40.2)	46(32.2)		47(39.8)	40(31.5)	
Coronary heart disease	62(41.1)	30(31.9)		42(41.2)	50(35.0)		51(43.2)	41(32.3)	
Hyperlipemia	71(47.0)	36(38.3)		47(46.1)	60(42.0)		47(39.8)	60(47.2)	
Previous stroke	28(18.5)	11(11.7)		20(19.6)	19(13.3)		21(17.8)	18(14.2)	
**Dysphagia**	71(47.7)	23(24.7)	#	52(51.0)	42(30.0)	#	60(50.8)	34(24.7)	#
**Functional assessment**									
BI	5[0–10]	10[5–40]	#	5[0–10]	5[5–20]	#	5[5–10]	5[5–20]	#
mRS	5[4–5]	4[4–5]	#	4[4–5]	4[4–5]	§	5[4–5]	4[4–5]	#
TCT	12[0–36]	48[24–61]	#	12[0–36]	36[12–48]	#	12[0–36]	36[12–48]	#
SBS	2[1–3]	3[2–3]	#	2[1–2]	2[2–3]	#	2[1–3]	2[2–3]	§
SPMSQ	7[5–10]	4[1–10]	#	7[5–10]	5[2–10]	#	7[5–10]	5[2–10]	#
**Laboratory parameters**									
Albumin, g/dL	2.88±0.37	3.70±0.26	#	2.76±0.39	3.51±0.35	#	2.85±0.46	3.5±0.34	#
Cholesterol, mg/dL	150.7±40.6	147.7±44.4		143.8±38.9	154.0±43.9		137.0±37.3	161.5±42.9	#
Lymphocyte count/mL	1300[900–1800]	1700[1200–1900]		1050[800–1300]	1700[1400–2000]	#	1100[800–1450]	1700[1400–2000]	#
Neutrophil count/mL	5600[4300–7525]	4800[3950–6200]	*	5650[4225–7275]	5100[4100–6700]		5600[4300–7550]	5050[4075–6500]	*
Hemoglobin, g/dL	12.6±1.5	13.3±1.7	§	12.3±1.5	13.4±1.5	#	12.4±1.6	13.3±1.5	#
Platelet count/mL × 1000	260[260–331]	244[194–296]		257[192–325]	246[209–304]		251[191–313]	2450[213–307]	
C-reactive protein, mg/L	23.6[9.7–57.1]	5.80[2.65–15.6]	#	29.5[9.8–81.7]	8.9[3.6–21.5]	#	22.5[8.9–67.0]	9.0[3.4–22.6]	#
Fibrinogen, mg/dL	566±176	480±133	#	565±193	511±141	§	561±194	509±132	§
D-dimer, μg/mL	1.40[0.81–2.91]	0.55[0.28–1.43]	#	1.77[0.92–3.26]	0.68[0.36–1.46]	#	1.50[0.81–3.09]	0.68[0.37–1.78]	#

Continuous variables were expressed as mean ± SD or median [interquartile range]. Categorical variables are expressed as frequencies (percentages). * *p* < 0.05; § *p* < 0.01; # *p* < 0.001. BMI = body mass index; BI = Barthel index; mRS = modified rankin scale; TCT = Trunk control test; SBS = sitting balance scale; SPMSQ = short portable mental status questionnaire; GNRI = geriatric nutritional risk index; PNI = prognostic nutritional index; CONUT = controlling nutritional status score.

**Table 3:** Partial correlations (adjusted for sex) of nutritional screening with general characteristics and functional assessment in stroke patients (n = 245).

	GNRI		PNI		CONUT Score	
	*r*	*p*	*r*	*p*	*r*	*p*
Age (years)	−0.270	<0.001	−0.328	<0.001	0.327	<0.001
BMI (kg/m^2^)	0.099	0.124	0.087	0.175	−0.079	0.221
BI	0.370	<0.001	0.319	<0.001	−0.271	<0.001
mRS	−0.352	<0.001	−0.303	<0.001	0.267	<0.001
TCT	0.452	<0.001	0.389	<0.001	−0.335	<0.001
SBS	0.356	<0.001	0.319	<0.001	−0.252	<0.001
SPMSQ	−0.327	<0.001	−0.258	<0.001	0.232	<0.001
C-reactive protein, mg/L	−0.485	<0.001	−0.479	<0.001	0.428	<0.001
Fibrinogen, mg/dL	−0.226	<0.001	−0.208	<0.001	0.220	<0.001
D-dimer, μg/mL	−0.431	<0.001	−0.494	<0.001	0.346	<0.001

GNRI = geriatric nutritional risk index; PNI = prognostic nutritional index; CONUT = controlling nutritional status score; BMI = body mass index; BI = Barthel index; mRS = modified rankin scale; TCT = Trunk control test; SBS = sitting balance scale; SPMSQ = short portable mental status questionnaire.

**Table 4:** Partial correlations (adjusted for sex) of nutritional screening with general characteristics and functional assessment in stroke patients with age ≥75 years (n = 105).

	GNRI		PNI		CONUT	
	*r*	*p*	*r*	*p*	*r*	*p*
Age (years)	−0.081	0.412	−0.050	0.615	0.050	0.613
BMI (kg/m^2^)	0.016	0.871	0.024	0.810	−0.035	0.801
BI	0.276	<0.01	0.263	<0.01	−0.275	<0.01
mRS	−0.255	0.013	−0.212	0.041	0.179	0.084
TCT	0.279	<0.01	0.210	0.045	−0.178	0.090
SBS	0.233	0.029	0.192	0.073	−0.187	0.081
SPMSQ	−0.189	0.085	−0.097	0.379	0.108	0.327
C-reactive protein, mg/L	−0.564	<0.001	−0.527	<0.001	0.513	<0.001
Fibrinogen, mg/dL	−0.337	<0.001	−0.307	<0.001	0.293	<0.001
D-dimer, μg/mL	−0.363	<0.001	−0.383	<0.001	0.296	<0.001

GNRI = geriatric nutritional risk index; PNI = prognostic nutritional index; CONUT = controlling nutritional status score; BMI = body mass index; BI = Barthel index; mRS = modified rankin scale; TCT = Trunk control test; SBS = sitting balance scale; SPMSQ = short portable mental status questionnaire.

## Data Availability

The data presented in this study are available on request from the corresponding author. The data are not publicly available due to privacy.
